# Supplementary material for: Myosin-dependent cell-cell communication controls synchronicity of division in acute and chronic stages of Toxoplasma gondii
Source: Nat Commun. 2017 Jun 8;8:15710. doi: 10.1038/ncomms15710 (PMC5477499; doi:10.1038/ncomms15710)
Supplement: Supplementary Information — Supplementary Figures, Supplementary Tables, Supplementary Methods and Supplementary References [file ncomms15710-s1.pdf]

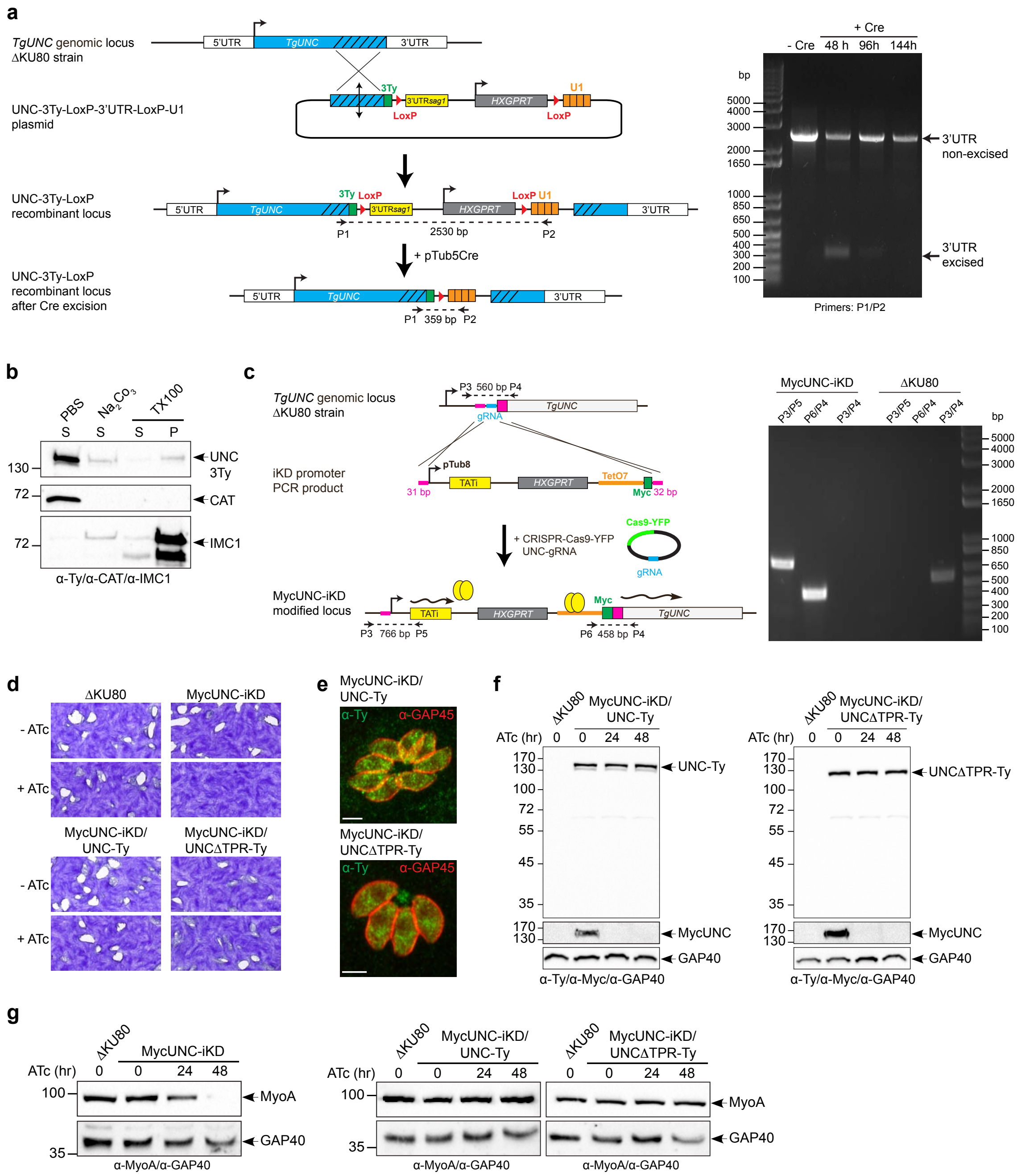

Supplementary Figure 1. Strategy used to knockdown and functionally complement TgUNC. (a) Schematic representation of the U1-based strategy used to tag endogenous TgUNC and to destabilize the mRNA upon Cre-recombinase excision (left panel). The PCRs performed on gDNA show that the excised parasites are rapidly lost after few passages (right panel). The primers used are listed in table S2. (**b**) The solubility of UNC-3Ty was evaluated by sequential extraction in PBS, Na2CO3 and Triton-X100. The cytoplasmic catalase (CAT) and the cytoskeleton protein IMC1 were used as controls for the soluble and insoluble fraction, respectively. S: soluble, P: pellet. (**c**) Schematic representation of the strategy used to replace the endogenous promoter of TgUNC by a Tet-inducible promoter (left panel) and the PCRs performed on gDNA extracted from a clone and showing the correct integration of the construct (right panel). Primers are listed in table S2. (**d**) Both full length TgUNC (UNC-Ty) or a mutant lacking the TPR domain (UNCΔTPR-Ty) are able to complement the growth defect of TgUNC depletion as monitored by plaque assay fixed after 7 days ± ATc. (**e**) UNC-Ty and UNCΔTPR-Ty localize to the cytoplasm of intracellular tachyzoites. Scale bars: 2 µm. (f) Total extract of extracellular tachyzoites expressing UNC-Ty and UNCΔTPR-Ty were subjected to immunoblot using α-Ty antibodies and migrated to their expected size while MycUNC-iKD was still regulatable by ATc. GAP40 was used as loading control. (**g**) Complementation of MycUNC-iKD by UNC-Ty or UNCΔTPR-Ty prevented the destabilization of TgMyoA. GAP40 was used as loading control.

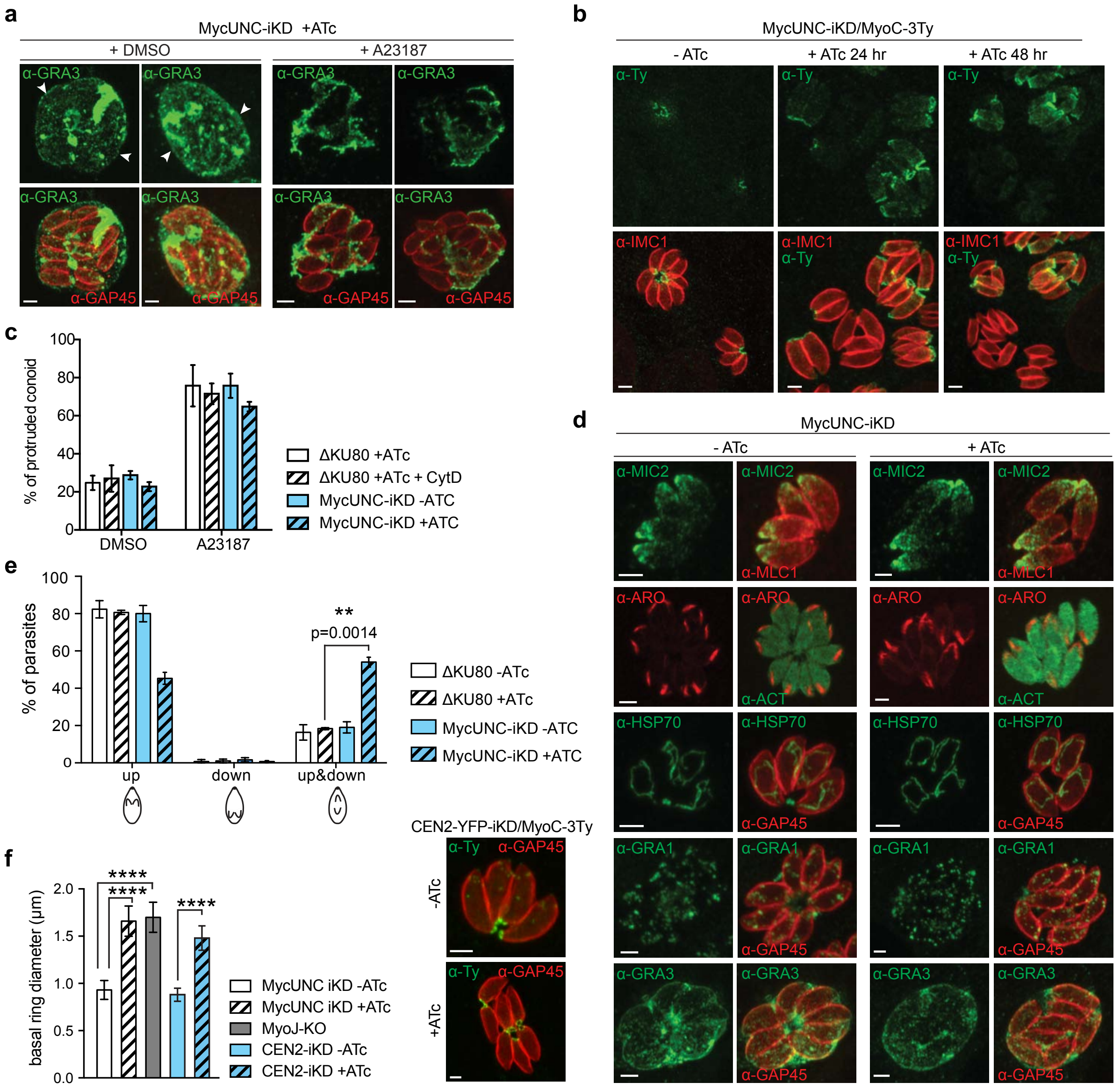

Supplementary Figure 2. Phenotypic analysis of TgUNC depletion. (**a**) IFA carried out after Ca2+ ionophore-induced egress of MycUNC-iKD parasites performed by treatment with DMSO or A23187 for 7 min after 56 hr of ATc treatment. The dense granule staining of GRA3 shows that the parasitophorous vacuole membrane is intact (arrowheads) with DMSO and disrupted upon A23187 treatment but the parasites visualized with α-GAP45 antibodies failed to egress. Scale bars: 2 μm. (**b**) IFA of intracellular MycUNC-iKD parasites expressing endogenously tagged TgMyoC-3Ty. The relocalization of TgMyoC along the periphery of the parasites is visible at 24 and 48 hr of ATc treatment, as well as the enlargement of the basal ring. At 48 hr of treatment, TgMyoC starts to disappear. (**c**) Conoid protrusion assay performed on ΔKU80 and MycUNC-iKD parasites treated ± ATc for 48 hours and ± Cytochalasin D (CytD) for 30 min. Data are represented as mean ± SD from 3 independent experiments. (**d**) The integrity and positioning of the organelles was checked in intracellular MycUNC-iKD parasites after 48 hr ± ATc. The micronemes (α-MIC2), rhoptries (α-ARO), mitochondrion (α-HSP70) and dense granules (α-GRA1 and α-GRA3) do not present any visible defect. The parasites were co-stained with α-ACT (cytoplasm), α-GAP45 or α-MLC1 (pellicle). Scale bars: 2 μm. (**e**) The orientation of the developing daughter cells (DC) was scored upon TgUNC depletion (48 hr of ATc) and compared to wild type and untreated parasites. The graph represents the percentage of DC growing in an up, down or up-and-down direction and is represented as mean ± SD (n=3). The significance of the results was assessed using a parametric paired t-test and the two-tailed p-value is written on the graph. (**f**) The size of the basal ring of the parasites has been measured on IFA pictures of MycUNC-iKD after 48 hr ± ATc (n=25 untreated and n=37 treated, as shown in fig. 2g) as well as in MyoJ-KO parasites (n=37, as shown in fig. 4a) and CEN2-iKD after 48 hr ± ATc (n=32 untreated and n=34 treated, right panel) using the signal of the endogenously tagged MyoC-3Ty. The significance of the results was assessed using a parametric paired t-test and the two-tailed p-values are <0.0001.

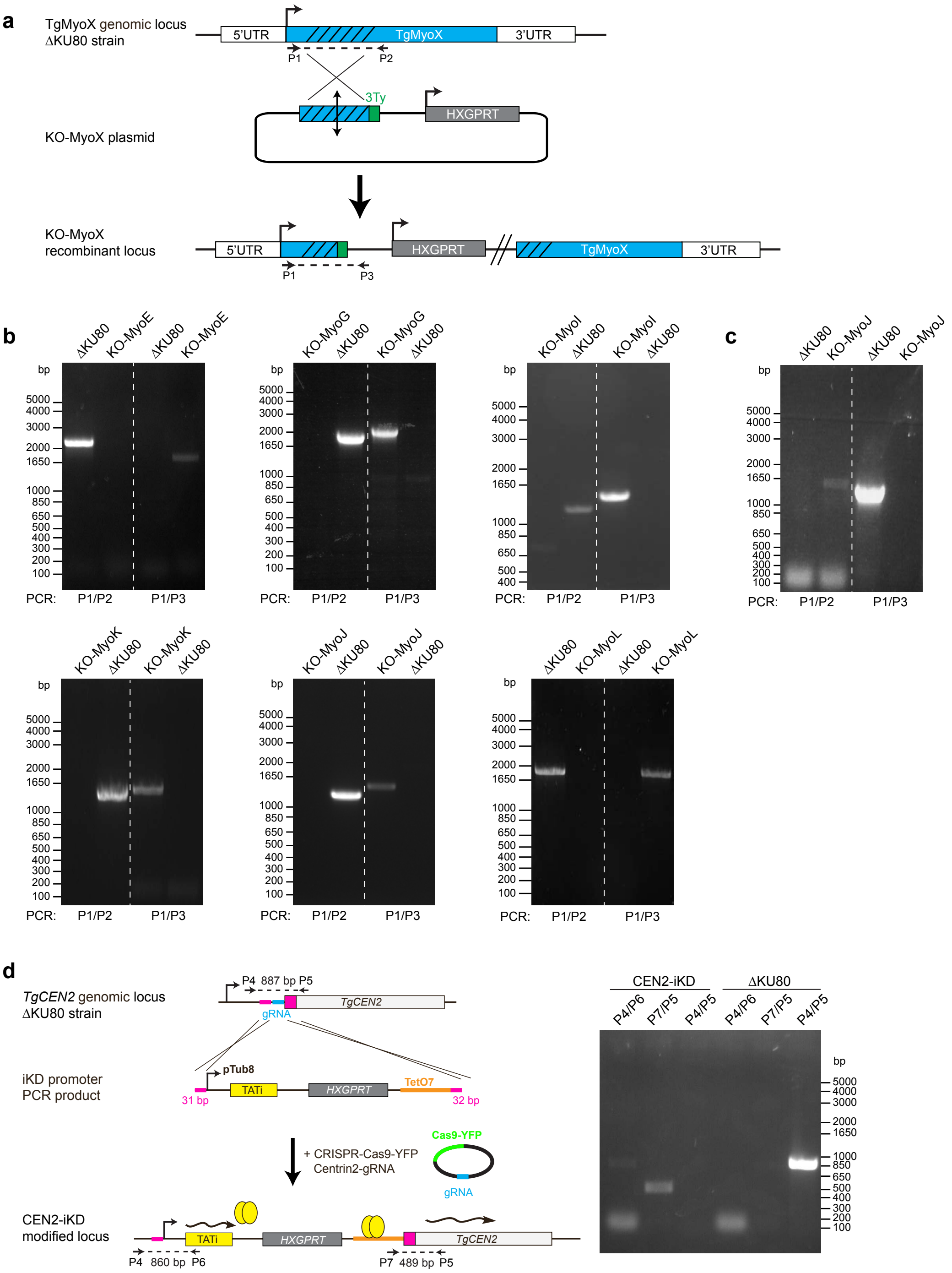

Supplementary Figure 3. Generation of myosin knockout strains. (**a**) A knockout of each uncharacterized myosin (TgMyoE, G, I, J, K, and L) has been generated in the ΔKU80 background, using a knock-in strategy consisting in recombining in the head domain in order to create a truncated and hence non-functional protein. (**b**) PCR analysis performed on gDNA extracted from stable clones confirms the correct integration of the constructs and the clonality of the strains. Primers are listed in table S2 and their position is indicated on the scheme (**a**). (**c**) PCR analysis performed on gDNA extracted from one stable clone to confirm the deletion of MyoJ (same construct as depicted in (**a**) except that the HXGPRT cassette has been replaced by a DHFR cassette) in the MyoI-KO background. (**d**) Schematic representation of the strategy used to replace the endogenous promoter of TgCEN2 by a Tet-inducible promoter (left panel) and the PCRs performed on gDNA extracted from a clone showing the correct integration of the construct (right panel). Primers are listed in table S2.

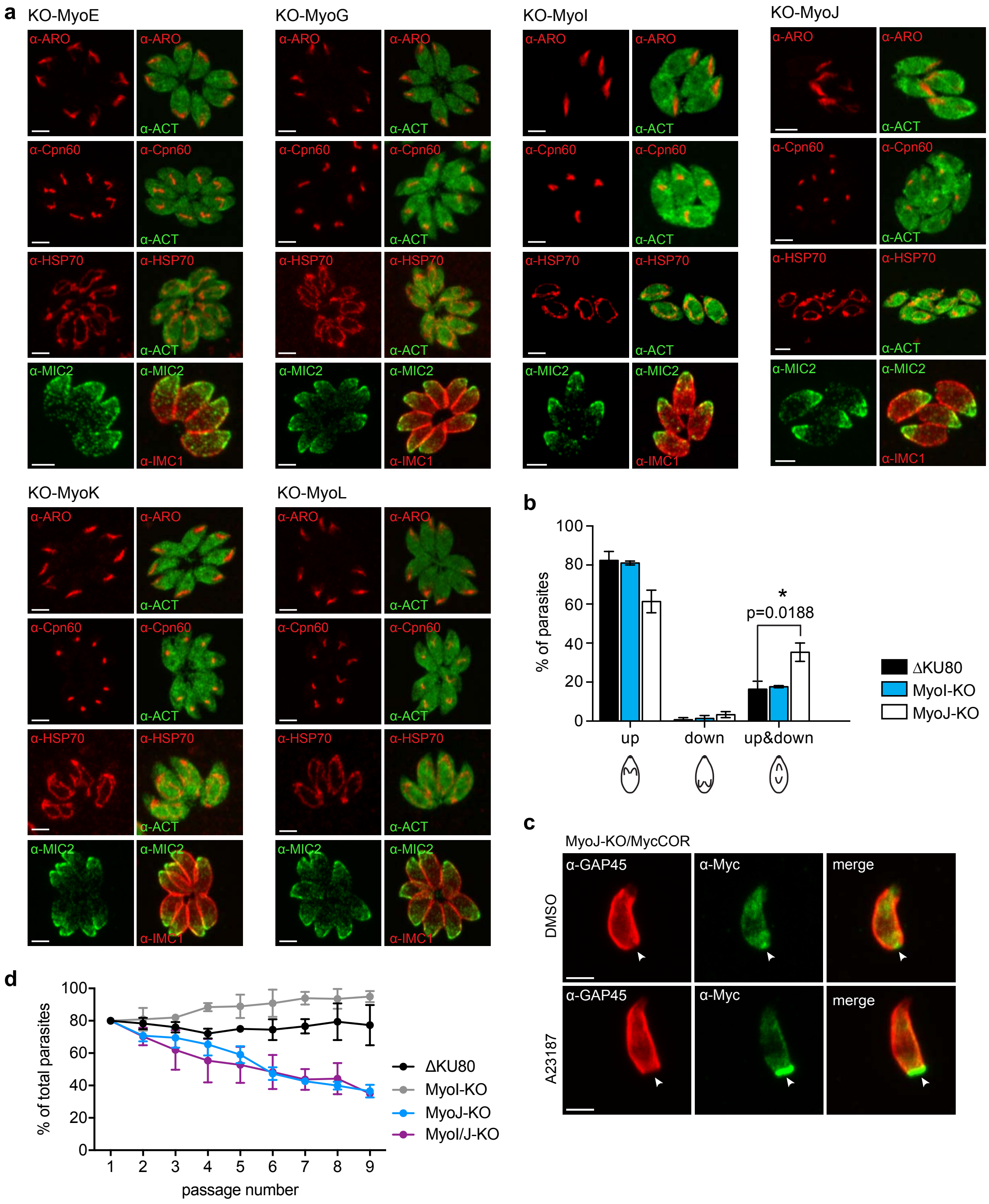

Supplementary Figure 4. Impact of myosin(s) deletion on organelles and fitness. (**a**) The integrity and positioning of the organelles was checked in intracellular MyoE-, MyoG-, MyoI-, MyoJ-, MyoK- and MyoL-KO parasites. The rhoptries (α-ARO), apicoplast (α-Cpn60), mitochondrion (α-HSP70) and micronemes (α-MIC2) do not present any visible defect. The parasites were co-stained with α-ACT (cytoplasm) or α-IMC1 (pellicle). Scale bars: 2 µm. (**b**) The orientation of the developing daughter cells (DC) was scored in MyoI-KO and MyoJ-KO and compared to wild type parasites (ΔKU80). Only MyoJ-KO presented a minor defect in the orientation of the daughter cells. The graph represents the percentage of DC growing in an up, down or up-and-down direction and is represented as mean ± SD from 3 independent experiments. The significance of the results was assessed using a parametric paired t-test. The two-tailed p-value is written on the graph. (**c**) Endogenously tagged Coronin (MycCOR) still relocalizes to the basal end (arrowheads) of extracellular MyoJ-KO tachyzoites upon calcium-ionophore (A23187) stimulation. (**d**) Competition assay performed on ΔKU80, MyoI-KO, MyoJ-KO and MyoI/J-KO using GFP-expressing parasites as an internal control. The graphs are presented as mean ± SD (n=3).

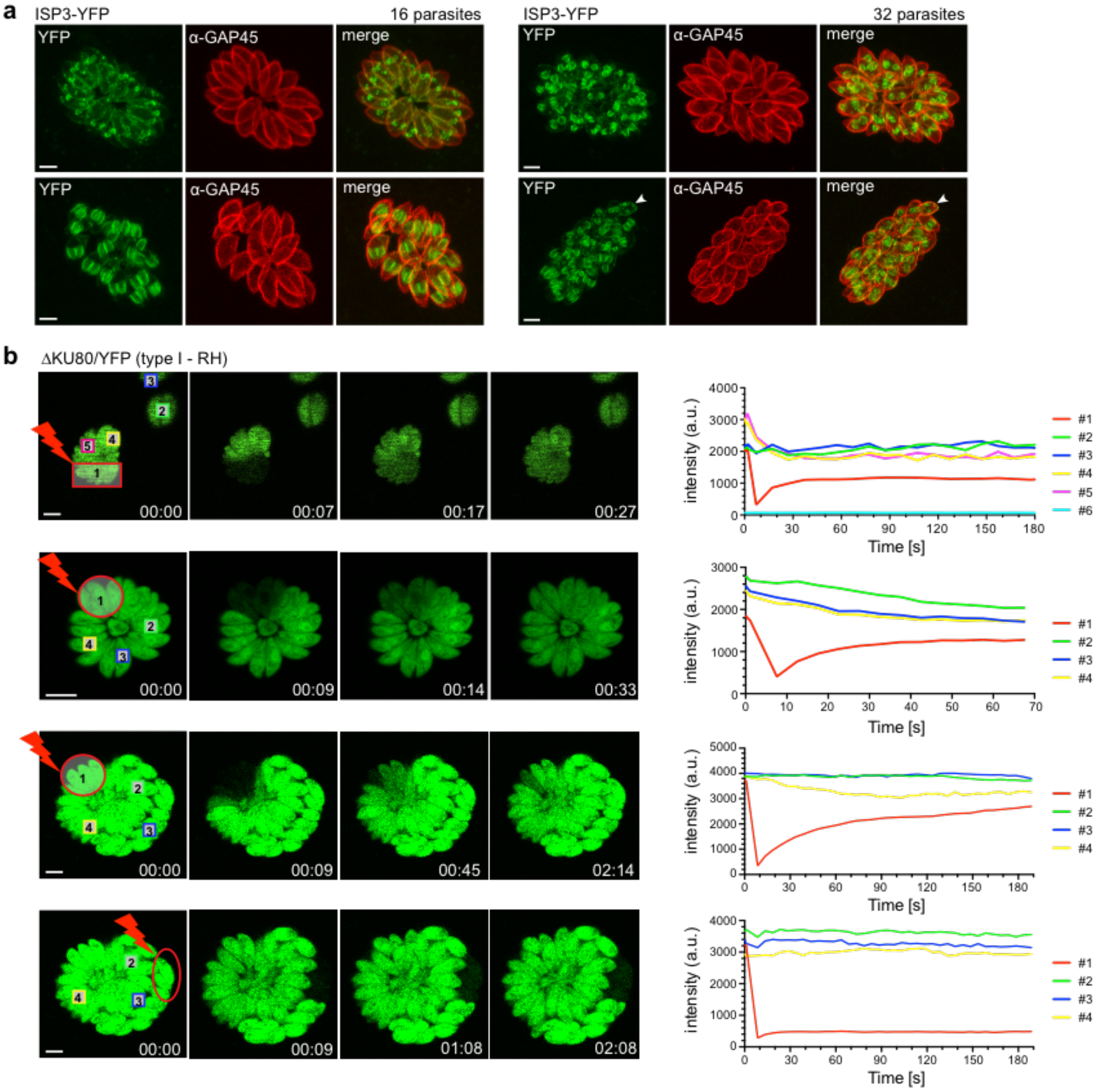

c

CytD treatment	Fluorescence recovery	Number of vacuoles
2-6 hours	yes	12 (homogenous)
	no	1
8-10 hours	yes	12 (homogenous: 7, non-homogeneous: 5)
	no	6

Supplementary Figure 5. The connection is occasionally lost in large vacuoles and is partially resistant to cytochalasin D. (**a**) ISP3 has been endogenously YFP-tagged to follow the daughter cells in large vacuoles (16 and 32 parasites). The synchronicity of division is usually observed in these vacuoles except in parasites (arrowhead) where the rosette organization is compromized. Scale bars: 2 µm. (**b**) The connection is preserved in large vacuoles of type I ΔKU80 parasites transiently expressing soluble GFP and the recovery of fluorescence in bleached parasites is faster when the number of connected parasites is higher where the rosette organization is compromized. Left panels: time-lapse imaging of the FRAP experiments. The bleached areas are delineated in red. Right panel: quantification of the intensity of GFP fluorescence recorded in the areas numbered on the left panel. Scale bars: 5 µm. (**c**) Quantification of FRAP experiments performed at 24 hr p.i. after short (2-6 hr) or long (8-10 hr) exposure to 1 µM of cytochalasin D (CytD). The recovery of fluorescence has been classified as homogeneous when the diffusion of GFP came from all the other parasites of the vacuole and non-homogeneous when the diffusion came from one or several but not all the other parasites of the vacuole.

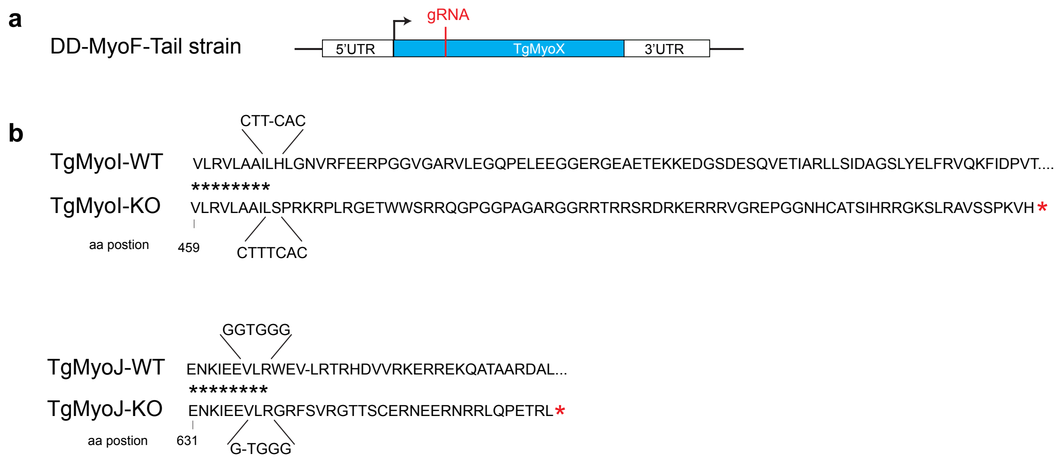

Supplementary Figure 6. Generation of DD-MyoF-tail/MyoI-KO and DD-MyoF-tail/MyoJ-KO. (**a**) The CRISPR/Cas9 strategy was used to disrupt the TgMyoI or TgMyoJ locus in the DD-MyoF-tail background using a sgRNA targeting the middle of the head domain in order to create a frameshift and therefore a truncated and non-functional protein. (**b**) Sequencing results of the clones used in this study with the position of the stop codon (red asterisk).

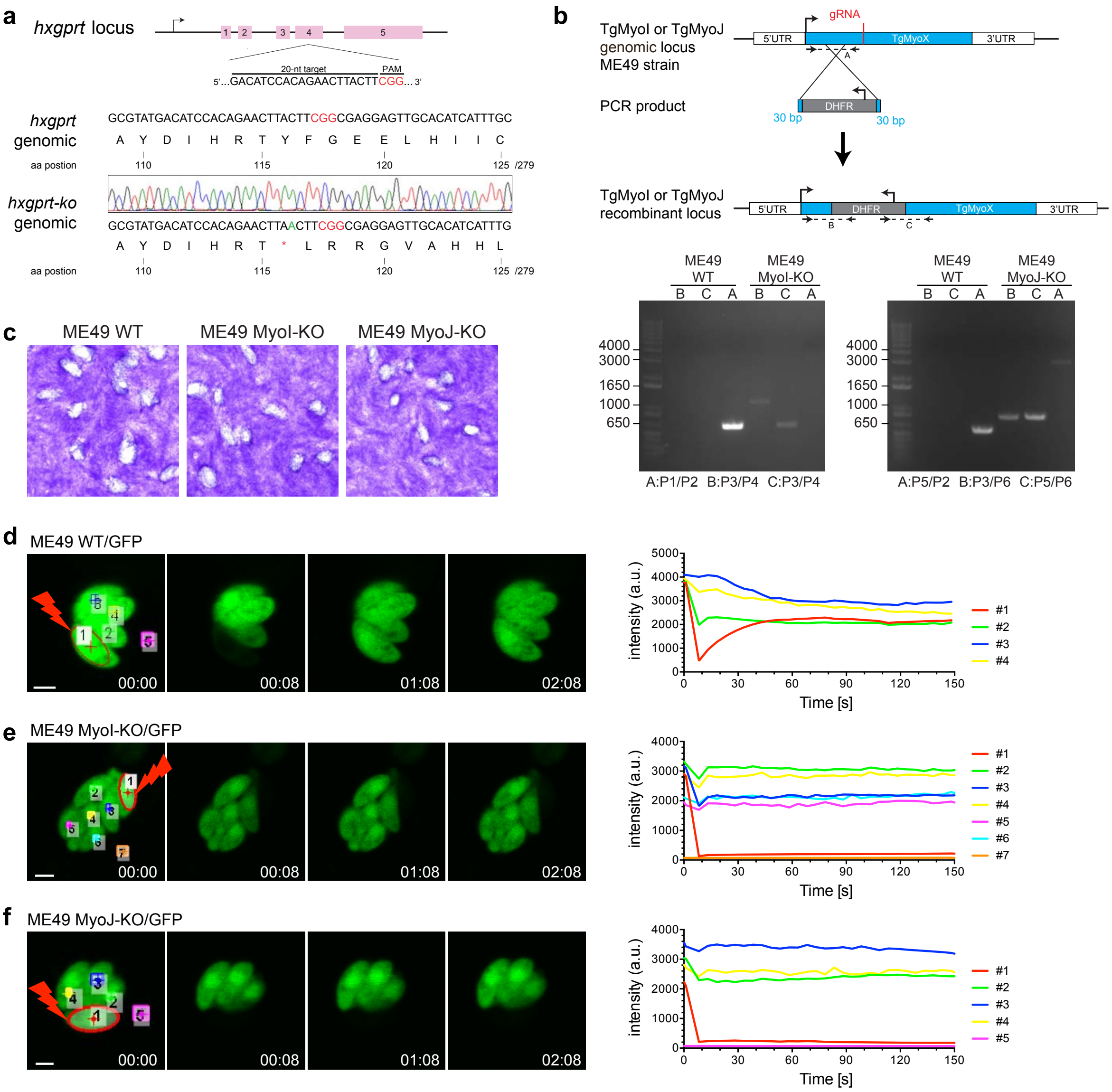

Supplementary Figure 7. The intravacuolar connection is conserved in type II ME49 tachyzoites and is dependent on TgMyoI and TgMyoJ expression. (**a**) Generation of the ME49Δhxgprt strain by using the CRISPR/Cas9 strategy to disrupt the hxgprt locus in the ME49 strain. (**b**) In the ME49Δhxgprt background, TgMyoI or TgMyoJ have been disrupted by insertion of a DHFR-TS cassette in the head domain (upper panel). The corresponding PCRs attesting of the correct insertion have been performed on gDNA with the primers listed in table S2. (**c**) The plaque assay performed on wild type (WT), MyoI-KO and MyoJ-KO over 10 days shows that MyoJ-KO parasites form slightly smaller plaques. (**d-f**) Left panels: time-lapse imaging of FRAP experiments performed on type II WT (**d**), MyoI-KO (**e**) and MyoJ-KO (**f**) parasites stably expressing soluble GFP. The bleached areas are delineated in red. Scale bars: 2 µm.Right panel: quantification of the intensity of GFP fluorescence recorded in the areas numbered on the left panel. The diffusion of soluble GFP is dependent of the presence of TgMyoI and TgMyoJ**.**

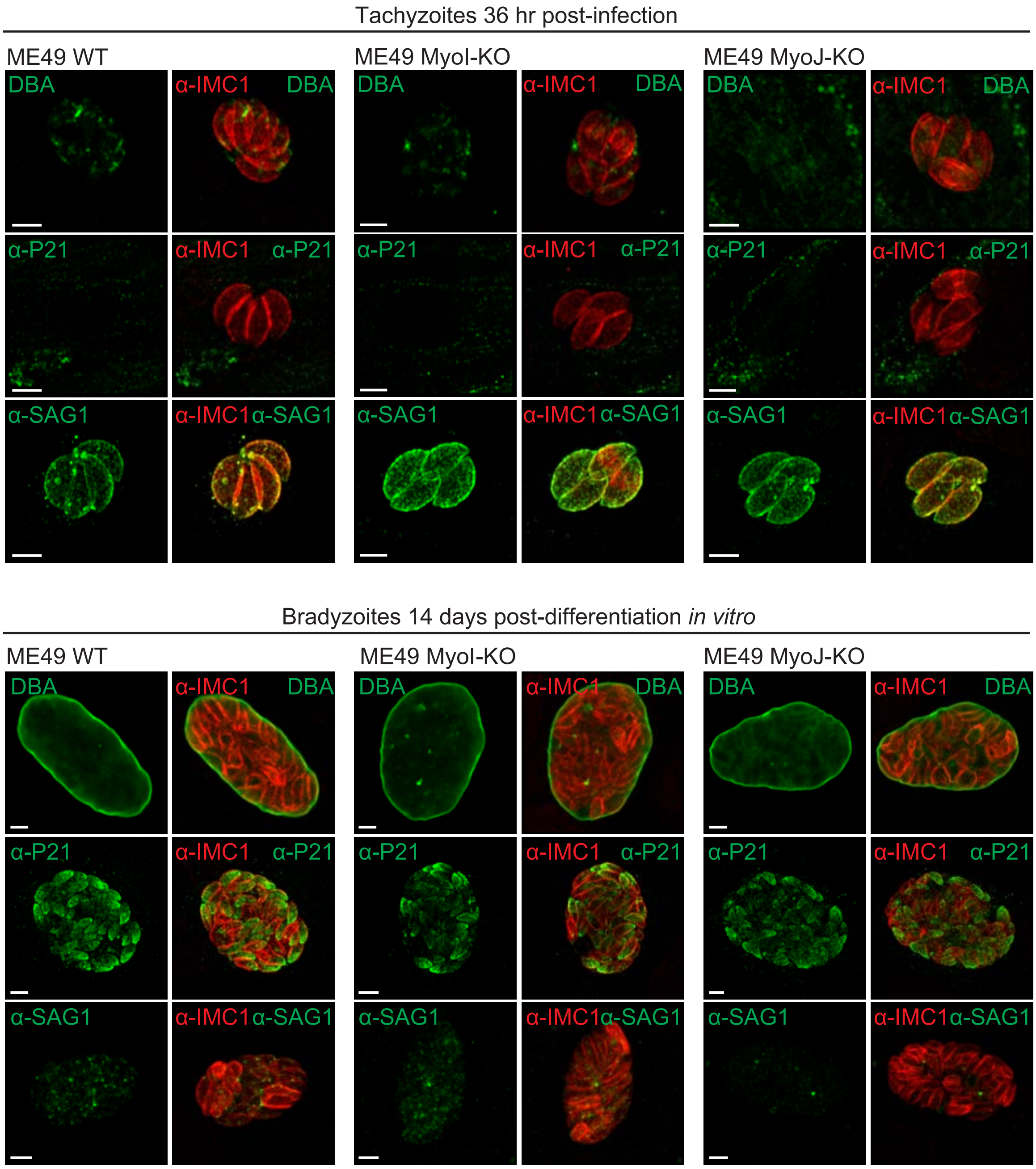

Supplementary Figure 8. Type II ME49 MyoI-KO and MyoJ-KO strains form normal cysts in vitro. Markers specific of the tachyzoite (SAG1) and bradyzoite (P21 and Dolichos Biflorus Agglutinin (DBA)) stages have been tested on tachyzoites 36 hr p.i. and on bradyzoites 14 days post-differentiation *in vitro*. No difference has been observed in MyoI-KO or MyoJ-KO compared to the parental cell line. Scale bars: 5 µm.

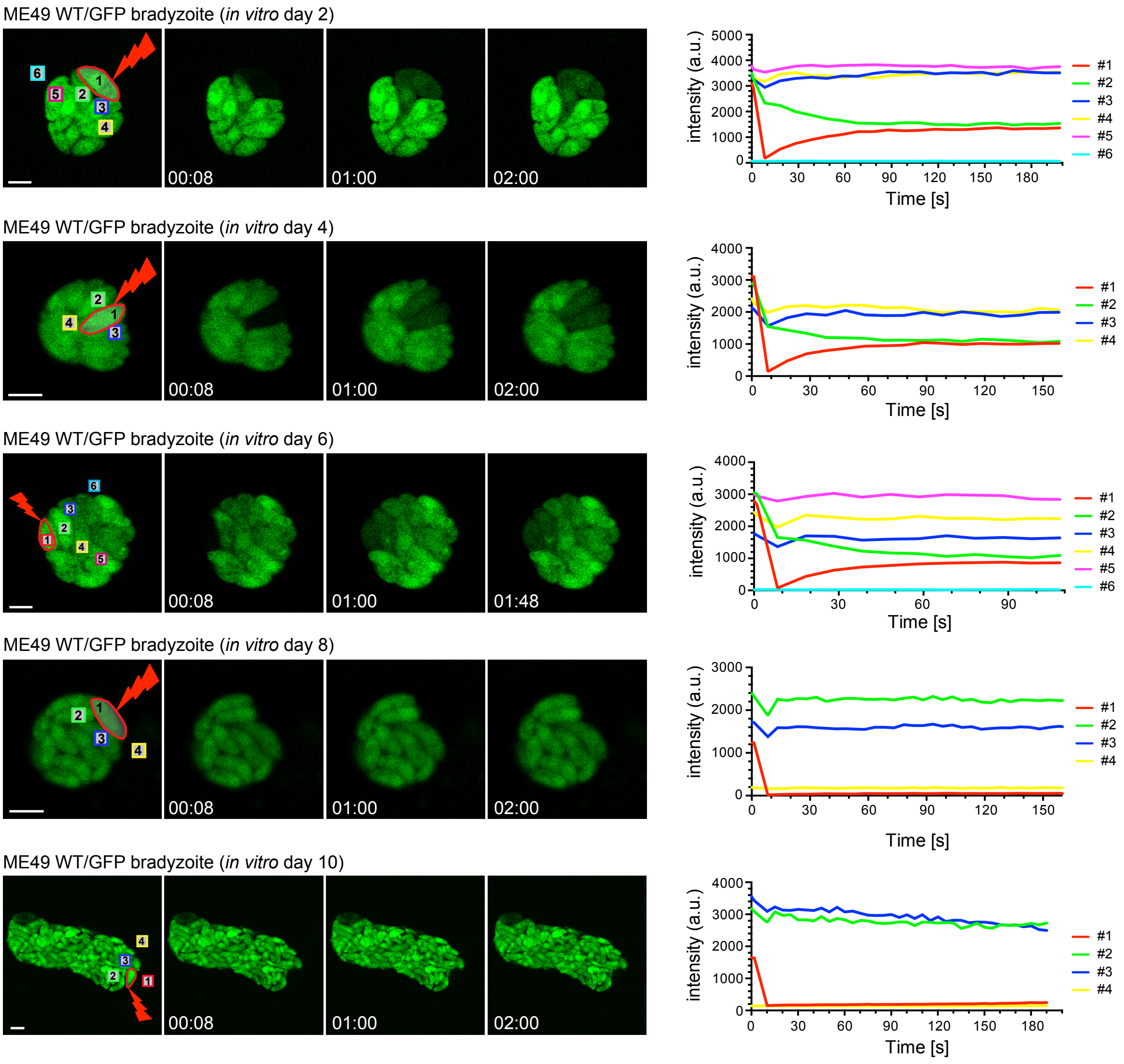

Supplementary Figure 9. Connection in type II ME49 parasites during in vitro differentiation. FRAP experiments performed every two days post-differentiation *in vitro* show that parasites are connected 2 by 2 up to day 6 and then start to be disconnected. Left panels: representative time-lapse imaging of FRAP experiments performed on ME49 WT parasites stably expressing soluble GFP. The bleached areas are delineated in red. Scale bars: 5 µm. Right panel: quantification of the intensity of GFP fluorescence recorded in the areas numbered on the left panels.

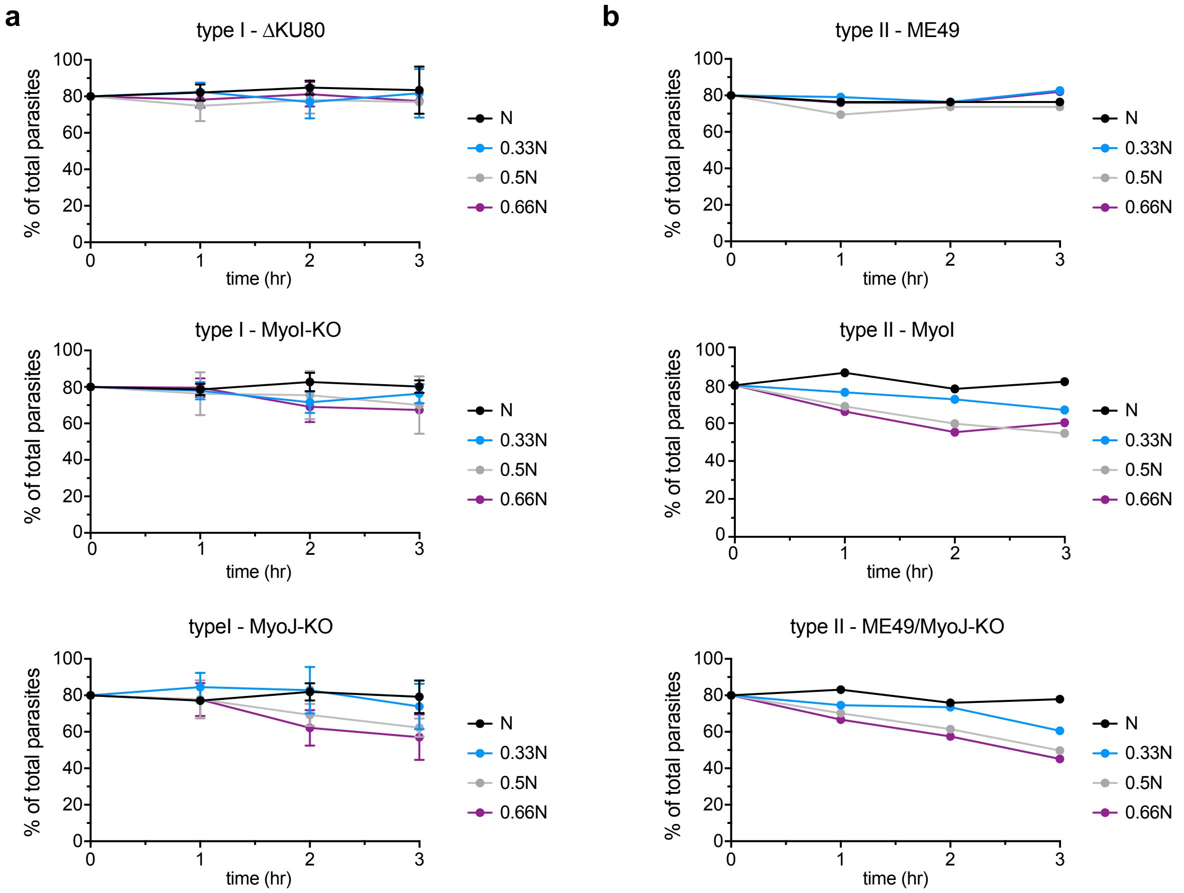

Supplementary Figure 10. Hypo-osmotic shock tested on type I and type II parasites. Extracellular parasites of type I (ΔKU80, MyoI-KO and MyoJ-KO) (**a**) or type II (ME49 WT, MyoI-KO and MyoJ-KO) (**b**) where mixed with GFP-expressing parasites and exposed for 1, 2 and 3 hr to different hypo-osmotic conditions, 0.33x, 0.5x and 0.66x normal osmotic strengh (by diluting the medium with water) before infecting new host cells. The ratio of the strain of interest vs GFP was then determined after 24 hr of intracellular growth. Results are presented as mean ± SD. (n=3 for type I strains and n=1 for type II strains).

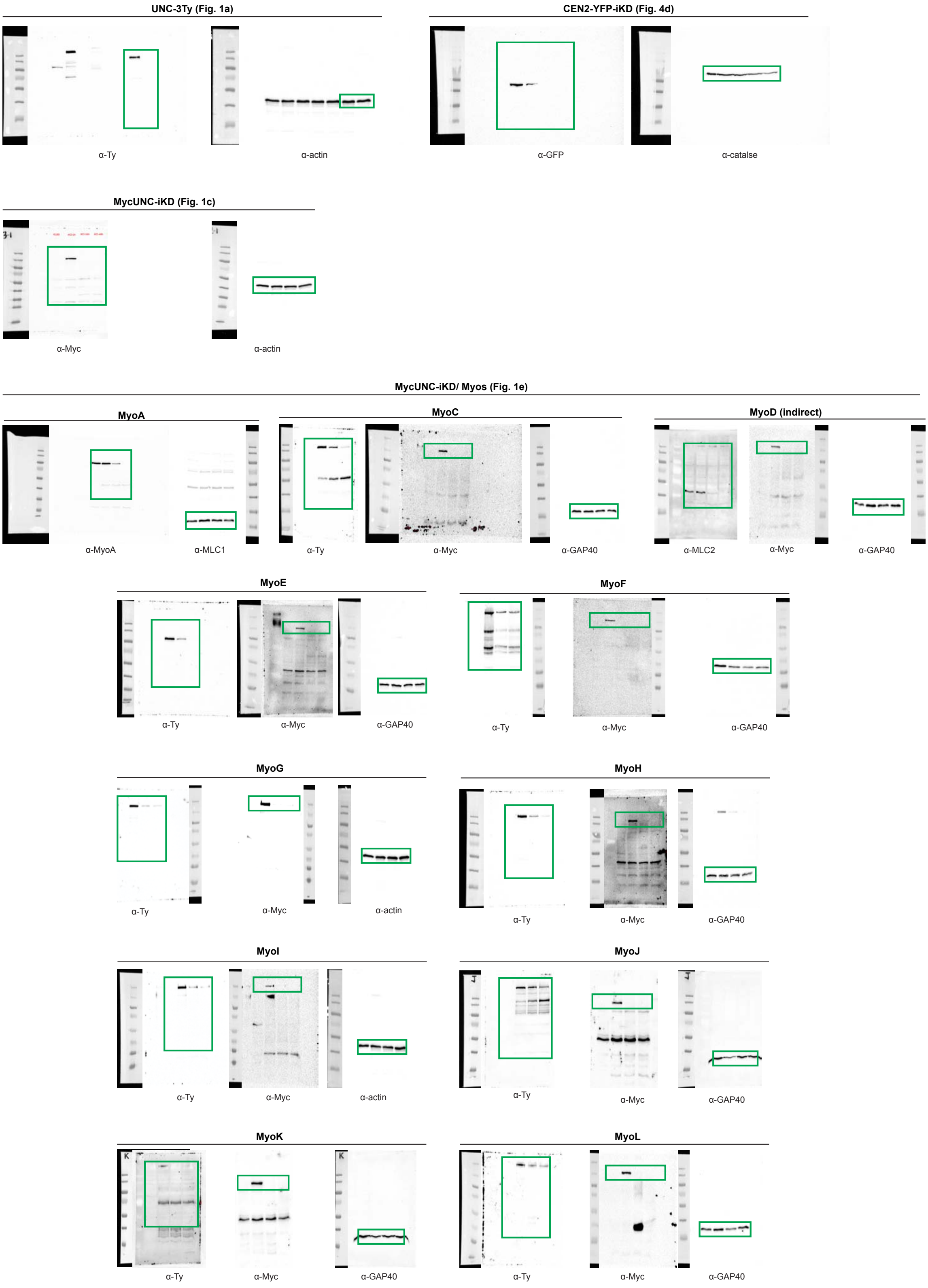

Supplementary Figure 11. Uncropped western blots of Figures 1a, 1c, 1e and 4d.

Supplementary Table 1. Oligonucleotides used in this study to generate the constructs. The underlined nucleotides correspond to the homology sequences.

Purpose	Restriction enzyme	Name and sequence of the oligonucleotide
KI-MyoE	*Kpn*I<br><br>*Nsi*I	TgMyoE-4355<br>CCGGTACCGAGCAGCTAGATCTCGAGGTCAC<br>TgMyoE-4356<br>CGGATGCATCCTCCATAATCACTCGCGCCTTCC
KO-MyoE	*Kpn*I<br><br>*Sbf*I	TgMyoE-4523<br>GCCGGTACCGAGGTGTCCACCACAATAGATAGG<br>TgMyoE-4354<br>GCGCCTGCAGGGGCCTTCGCTAAGGAGTCCCG
KI-MyoG	*Kpn*I<br><br>*Nsi*I	TgMyoG-2746<br>CCGGTACCTATGGGTGTAGTTTTTAGCACAGG<br>TgMyoG-2747<br>GGCATGCATCACCTGTCGGAGGAGCCTG
KO-MyoG	*Kpn*I<br><br>*Nsi*I	TgMyoG-4237<br>GCGGGTACCGCGTTGGCTTGCGTATCCCTTGTC<br>TgMyoG-4238<br>GCGATGCATGGCCCAAGAGCTCGCTCGCTG
KI-MyoI	*Mfe*I<br><br>*Sbf*I	TgMyoI-2758<br>CCGCAATTGCGCAAAAAGTGGAAAACTGTTGTGAAATATCTC<br>TgMyoI-2759<br>CCGCCTGCAGGCCCCACTGGACGAATGTGCGCG
KO-MyoI	*Kpn*I<br><br>*Nco*I	TgMyoI-4017<br>GCGGGTACCGAACGCGGCGAAGCAGAGACAGAAAAG<br>TgMyoI-4018<br>GCGCCATGGGAGAAGATATGGTGGTTGAAGTGCTGTTGAAGTTTC
KI-MyoJ	*Kpn*I<br><br>*Nsi*I	TgMyoJ-4289<br>GCGGTACCGTGTGAGAATGGGGACAGGAG<br>TgMyoJ-4290<br>CGGATGCATCCATTTCCAGCTGCGACCATGCTTC
KO-MyoJ	*Kpn*I<br><br>*Nsi*I	TgMyoJ-4291<br>CCGGTACCGAAGAAGTTCTGCGGTGGGAGGTTC<br>TgMyoJ-4292<br>CCGATGCATGTGTCACTCTTCGCGGCGAAC
KI-MyoK	*Kpn*I<br><br>*Nsi*I	TgMyoK-4285<br>GGGGTACCGTGAGAGAGGAAAGCGACCGT<br>TgMyoK-4286<br>CCAATGCATGCTGCGCCGCCGGGAAGTTAG
KO-MyoK	*Kpn*I<br><br>*Nsi*I	TgMyoK-4287<br>GGGGTACCCGAAGCTGCGCCAGGACATCT<br>TgMyoK-4288<br>CCAATGCATCGCAAAGTAGTCCACTTGTCTG
KI-MyoL	ApaI<br><br>*Sbf*I	TgMyoL-5549<br>ATTGGGCCCCAAGTGGATCGAACAAGAAAAGCC<br>TgMyoL-5548<br>ATTCCTGCAGGCTTGCTTGAATCTGATCCTAAATTTG
KO-MyoL	*Kpn*I<br><br>*Nsi*I	TgMyoL-5648<br>CCCGGTACCGCGCAGATGACTTTTCAGCTTTATCG<br>TgMyoL-5649<br>CCCATGCATGAGTTTGCCCTCGAGATCTTGTGG

KI-UNC	*Kpn*I<br><br>*Nsi*I	TgUNC-5076<br>GTCGGTACCAGAGCGCGAATGTCGGGTG<br>TgUNC-5077<br>GACATGCATGGCTTGAGTCTGGGGTGGATTC
UNC-iKD PCR for transfection		TgUNC-5364<br>CAGTTTTGAGTTGTGCACCTGTTTCCAGTGCATGTTTGCGGATCCGGGG<br>TgUNC-5365<br>GCGAGCTAACGTAGCGTTTGACAAATCCTCCAGGTCCTCCTCGGAGATGA
gRNA-UNC		TgUNC-5366<br>GACCGTCAAATATACACATCAGTTTTAGAGCTAGAAATAGC<br>gRNA-rev-4883<br>AACTTGACATCCCCATTTAC
UNC-iKD WT complemen-tation	*Mfe*I<br><br>*Nsi*I	TgUNC-5689<br>GCGCAATTGCCGTCAAATATACACATCATGGAG<br>TgUNC-5077<br>GACATGCATGGCTTGAGTCTGGGGTGGATTC
UNC-iKD ΔTPR complemen-tation	*Mfe*I<br><br>*Nsi*I	TgUNC-5690<br>GCGCAATTGCGACAAAATGGAGATGCTCGCGGGGGTTC<br>TgUNC-5077<br>GACATGCATGGCTTGAGTCTGGGGTGGATTC
gRNA-MORN1		TgMORN1-5608<br>GTTCCTTACCGTTGTCCACCAGTTTTAGAGCTAGAAATAGC<br>gRNA-rev-4883<br>AACTTGACATCCCCATTTAC
MycGFP-MORN1 PCR for transfection		TgMORN1-5609<br>ACTTCATTCCCCGTTTTTCCTTACCGTTGCGACAAAATGCAGGAGCAGAA<br>TgMORN1-5610<br>CTGTCCGTGGTACGCGTGGCAGCTCTCTGCAGCTTTGTATAGTTCATCCA
gRNA MyoI-KO		TgMyoI-6021<br>GACGTTTCCGAGGTGAAGAAGTTTTAGAGCTAGAAATAGC<br>gRNA-rev-4883<br>AACTTGACATCCCCATTTAC
MyoI-KO PCR for transfection (DHFR)		TgMyoI-6022<br>CACACACCTTGGCTTCTCACAGGAAGAACGGCGGCCGCTCTAGAACTAG<br>TgMyoI-6023<br>TTCGCCGCGTTCTCCTCCCTCCTCGAGCTCGCGGAAGATCCGATCTTGC
gRNA MyoJ-KO		TgMyoJ-6024<br>GATCGAAGAAGTTCTGCGGTGTTTTAGAGCTAGAAATAGC<br>gRNA-rev-4883<br>AACTTGACATCCCCATTTAC
MyoJ-KO PCR for transfection (DHFR)		TgMyoJ-6025<br>GACGACGGCCTCGAAAGTGGCGGGGAGAAGGCGGCCGCTCTAGAACTAG<br>TgMyoJ-6026<br>CCGGCATCTGTCTCTTCCTTGGCGCACTTGGCGGAAGATCCGATCTTGC
gRNA CDPK2-KO		TgCDPK2-6016<br>GAATGAAGGCGGAGCGTTTGAGTTTTAGAGCTAGAAATAGC<br>gRNA-rev-4883<br>AACTTGACATCCCCATTTAC
CDPK2-KO PCR for transfection (DHFR)		TgCDPK2-6019<br>GCCACTCCATCAGTACGCAAGCTGCGGACGGCGGCCGCTCTAGAACTAG<br>TgCDPK2-6020<br>TGTAGTAGTTTGTGATGGGACCAGTGTTGGGCGGAAGATCCGATCTTGC
gRNA HXGPRT-KO		gRNA-HX-5950<br>GACATCCACAGAACTTACTTGTTTTAGAGCTAGAAATAGC<br>gRNA-rev-4883<br>AACTTGACATCCCCATTTAC

CEN2-iKD PCR for transfection		TgCEN2-6314<br>TCCCAGTGTAAAATAAGAGGCGTGTATTGGCATGTTTGCGGATCCGGGG<br>TgCEN2-6315<br>GCTCGCCCCTCGCAGTGCTCCTCGCTGCATTTTGATATCCCTAGGAATTC
gRNA-CEN2		TgCEN2-6313<br>GTGGAGAGGAAAGGTGCGTTTGTTTTAGAGCTAGAAATAGC<br>gRNA-rev-4883<br>AACTTGACATCCCCATTTAC
GFP-SET8	*Eco*RI<br><br>*Bam*HI	SET8-6989<br>GCGGGATCCAGTAAAGGAGAAGAACTTTTCACTG<br>SET8-6990<br>GCGGAATTCTTTGTATAGTTCATCCATGCCATG

Supplementary Table 2. Primers used in this study to screen integration of the constructs.

Figure	Purpose	Name and sequence of the oligonucleotide
Figure S1	KI-UNC-3Ty	TgUNC-5246 (P1)<br>GGAAGTCCTCACGGCAGAACTC<br>M13-Fwd (P2)<br>GTAAAACGACGGCCAGT
	MycUNC-iKD	TgUNC-5498 (P3)<br>TTTCAGCCGTTGCGTCAGAAGT<br>TgUNC-5476 (P4)<br>TCTTCACGTAGGCCGGATGG<br>tetR-2903 (P5)<br>GAGCGAGTTTCCTTGTCGTCAGGCC<br>SAG1-1935 (P6)<br>CGCTGCACCACTTCATTATTTCTTCTGG
Figure S3	KO-MyoE	TgMyoE-4419 (P1)<br>GGCATCGAACTGTCGTATGTTTTG<br>TgMyoE-350 (P2)<br>GAGAAGCTGTTCCAGGGA
	KO-MyoG	TgMyoG-4482 (P1)<br>GATTTGGGAAGTGGATTGAAGTC<br>TgMyoG-5624 (P2)<br>TCCACCGCAGACACCTACCG
	KO-MyoI	TgMyoI-4130 (P1)<br>CGAACTTCCAAGAGCTCCTCGC<br>TgMyoI-5533 (P2)<br>GCGGGTCCTTCTGGATGTGG
	KO-MyoJ	TgMyoJ-4361 (P1)<br>GAGGCGCATTCGCAGTCAGAG<br>TgMyoJ-4362 (P2)<br>GCGAGTTGGACGAAATGCCTTC
	KO-MyoK	TgMyoK-4349 (P1)<br>GTGAGGCCTCTGAATGCATGCG<br>TgMyoK-4350 (P2)<br>ACGCAGATCGCCTGTTAGAGC
	KO-MyoL	TgMyoL-5697 (P1)<br>GGACCGAATTCAGTCTTTAGAGAAATG<br>TgMyoL-5698 (P2)<br>GTCGAAGGTCTTGAATCTCTCCG
	KO Myos	P30A-3980 (P3)<br>GTGACACCTGCAAGCCACAGCGG
	CEN2-iKD	TgCEN2-6317 (P4)<br>CTGCAGCTGTTGCTCGCTCGG<br>TgCEN2-6318 (P5)<br>CGAGAACATGAGGTGAGACGC<br>tetR-2903 (P6)<br>GAGCGAGTTTCCTTGTCGTCAGGCC<br>SAG1-1935 (P7)<br>CGCTGCACCACTTCATTATTTCTTCTGG
Figure S6	KO-MyoI	TgMyoI-6077 (P1)<br>GAACGCGACGCATATCGGCT<br>DHFR-2017 (P2)<br>GTCACTTGTTGTGCCAGTTCTAC<br>DHFR-2018 (P3)<br>CTTGGGGGTCATCGCGACGACCAGAC<br>TgMyoI-6078 (P4)<br>CTTGTTTGGTCACAGGGTCAATG
	KO-MyoJ	TgMyoJ-6079 (P5)<br>CTCTGAAGGCTGGAGACACTC<br>DHFR-2017 (P2)<br>GTCACTTGTTGTGCCAGTTCTAC<br>DHFR-2018 (P3)<br>CTTGGGGGTCATCGCGACGACCAGAC<br>TgMyoJ-6080 (P6)<br>CAACCTCTCCGAAGCCCTAC

Supplementary Table 3. Antibodies used in this study.

Antibody	Specie	WB dilution	IFA dilution	Reference/Company
α-MyoA	Rabbit	1:1000	-	[1]
α-MLC1	Rabbit	1:1000	1:1000	[1]
α-GAP40	Rabbit	1:1000	-	[2]
α-IMC1	Rabbit	1:1000	1:1000	[2]
α-GAP45	Rabbit	-	1:10000	[3]
α-MLC2	Rabbit	1:1000	-	[4]
α-Cpn60	Rabbit	-	1:1000	[5]
α-ISP1	Mouse	-	1:1000	[6]
α-ARO	Rabbit	1:1000	-	[7]
α-HSP70	Rabbit	-	1:1000	[8]
α-CAT	Rabbit	1:1000	-	[9]
α-MIC2 hybridoma supernatent	Mouse	-	1:10	Generous gift from J-F Dubremetz
α-GRA1 hybridoma supernatent	Mouse	-	1:10	Generous gift from J-F Dubremetz
α-GRA3 hybridoma supernatent	Mouse	-	1:10	Generous gift from J-F Dubremetz
α-P21 hybridoma supernatent	Mouse	-	1:50	Generous gift from J-F Dubremetz
α-ACT hybridoma supernatent	Mouse	1:4	1:10	[1]
α-SAG1 hybridoma supernatent	Mouse	-	1:10	Generous gift from J-F Dubremetz
α-Myc (mAb 9E10)	Mouse	1:200	1:200	Santa Cruz Biotechnology
α-Ty (mAb BB2) hybridoma supernatent	Mouse	1:4	1:10	[10]
α-rabbit IgG peroxydase	Goat	1:3000	-	Sigma
α-mouse IgG peroxydase	Goat	1:3000	-	Sigma
DBA-fluorescein		-	1:1000	Reactolab
Alexa Fluor 488 α-mouse IgG	Goat	-	1:3000	Thermofisher
Alexa Fluor 488 α- rabbit IgG	Goat	-	1:3000	Thermofisher
Alexa Fluo 594 α-mouse IgG	Goat	-	1:3000	Thermofisher
Alexa Fluo 594 α-rabbit IgG	Goat	-	1:3000	Thermofisher
Alexa Fluo 405 α-rabbit IgG	Goat	-	1:3000	Thermofisher

Supplementary methods

Subcellular fractionations

Freshly egressed parasites were harvested, washed in PBS and then resuspended in PBS and lysed by freeze and thaw. Pellet and soluble fractions were separated by centrifugation for 15 min at 14,000 rpm at 4°C. Supernatant was transferred into a new tube while the pellet was resuspended in PBS/0.1 M Na_2CO_3 [pH 11.5] and incubated at RT for 10 min. Pellet and soluble fractions were again separated by centrifugation for 15 min at 14,000 rpm at 4°C. Supernatant was transferred into a new tube while the pellet was resuspended in PBS/1% Triton X-100. Pellet and soluble fractions were again separated by centrifugation for 15 min at 14,000 rpm at 4°C. The soluble fractions were mixed with SDS–PAGE loading buffer under reducing conditions while the final pellet was resuspended into the loading buffer.

Orientation of daughter cell growth

Freshly egressed parasites were inoculated on confluent HFFs and allowed to grow for 24 hours before fixation with PFA/GA. For Myc-UNC-iKD parasites, a pre-treatment ± ATc was performed 24 hours before egress and continued for 24 hours before fixation with PFA/GA. To evaluate the orientation of the daughter cells, double-labeling IFA was performed using α-ISP1 and α-IMC1 antibodies. The up, down and up-and-up orientation was scored in parasites 100 parasites in duplicate for three independent experiments.

Conoid protrusion assay

Freshly egressed ΔKU80 and Myc-UNC-iKD parasites treated for 48 hours ± ATc were pelleted and resuspended in an HEPES/calcium-saline solution and stimulated either with calcium ionophore A23187 (3 μM) or DMSO. Parasites were then incubated for 8 min at 37°C on poly-L-lysine coated coverslips and fixed with PFA/GA. For cytochalasin D (CytD) treatment, parasites were pre-treated extracellularly for 30 min before doing experiment with 1 μM of CytD directly added in the medium of culture. The average number of protruded parasites was determined by counting 100 parasites for each condition for three independent experiments using the 63x magnification under DIC condition.

CytochalasinD (CytD) experiments

For FRAP experiments, intracellular parasites were treated 24 hours post infection with 1μM of CytD for 2, 6, 8 and 10 hours. The number of vacuole examined in each condition is mentioned in Supplementary Fig. 5c.

Measurement of the basal pole

The size of the basal ring of the parasites has been measured from multiple IFA pictures documented by confocal microscopy for MycUNC-iKD after 48 hours ± ATc (n=25 untreated

and n=37 treated), MyoJ-KO parasites (n=37), CEN2-iKD after 48 hours ± ATc (n=32 untreated and n=34 treated) and for MyoJ-3Ty/CEN2-YFP ± 1 µM CytD (n=25 untreated and 33 treated) using the signal of the endogenously tagged MyoC-3Ty. The diameter of the basal complex was measured using ImageJ and only in non-dividing parasites. Results are expressed as mean ± SD.

Osmotic shock assay

Extracellular parasites of type I (ΔKU80 and MyoJ-KO) (a) or type II (ME49 WT and MyoJ-KO) (b) where mixed with GFP-expressing parasites and exposed for 0, 1, 2 and 3 hours to different hypo-osmotic conditions: 0.33x, 0.5x and 0.66x normal osmotic strengh by diluting the medium with water, before infecting new host cells. The parasites were allowed to grow for 24 hours before fixing. A GAP45 staining was performed and 100 vacuoles were counted for three independent experiments for type I and for one experiment for type II. The ratio of the strain of interest vs GFP was normalized to 80% at t0. The data are presented as mean ± SD.

Supplementary references

1. Herm-Gotz, A. *et al*. *Toxoplasma gondii* myosin A and its light chain: a fast, single-headed, plus-end-directed motor. Embo J 21, 2149-2158 (2002).

2. Frenal, K., Marq, J. B., Jacot, D., Polonais, V. & Soldati-Favre, D. Plasticity between MyoC- and MyoA-glideosomes: an example of functional compensation in *Toxoplasma gondii* invasion. PLoS Pathog 10, e1004504 (2014).

3. Frenal, K. *et al*. Functional dissection of the apicomplexan glideosome molecular architecture. Cell Host Microbe 8, 343-357 (2010).

4. Polonais, V. *et al*. Unusual anchor of a motor complex (MyoD-MLC2) to the plasma membrane of *Toxoplasma gondii*. Traffic 12, 287-300 (2011).

5. Agrawal, S., van Dooren, G. G., Beatty, W. L. & Striepen, B. Genetic evidence that an endosymbiont-derived endoplasmic reticulum-associated protein degradation (ERAD) system functions in import of apicoplast proteins. J Biol Chem 284, 33683-33691 (2009).

6. Beck, J. R. *et al*. A novel family of Toxoplasma IMC proteins displays a hierarchical organization and functions in coordinating parasite division. PLoS Pathog 6, e1001094 (2010).

7. Mueller, C. *et al*. The Toxoplasma protein ARO mediates the apical positioning of rhoptry organelles, a prerequisite for host cell invasion. Cell Host Microbe 13, 289-301 (2013).

8. Pino, P. *et al*. Dual targeting of antioxidant and metabolic enzymes to the mitochondrion and the apicoplast of *Toxoplasma gondii*. PLoS Pathog 3, e115 (2007).

9. Ding, M., Clayton, C. & Soldati, D. *Toxoplasma gondii* catalase: are there peroxisomes in toxoplasma? J Cell Sci 113, 2409-2419 (2000).

10. Bastin, P., Bagherzadeh, Z., Matthews, K. R. & Gull, K. A novel epitope tag system to study protein targeting and organelle biogenesis in *Trypanosoma brucei*. Mol Biochem Parasitol 77, 235-239 (1996).
